# What a Doctor Should Do, and What He Should Not Do in Case of Labor*Read before the Palestine meeting, East Texas Medico-Chirurgical Society, May, 1902.

**Published:** 1902-12

**Authors:** T. J. Bell

**Affiliations:** Tyler, Texas


For Texas Medical Journal.

### What a Doctor Should Do, and What He Should Not Do in Case of Labor.*

BY T. J. BELL, M. D., TYLER, TEXAS.

A great deal has been written concerning the management of labor cases, men spin out long and elaborate theories as to the technique and management of labor cases, and insist on its being carried out in all cases, when in reality a large part of this theory is

*Read before the Palestine meeting, East Texas Medico-Chirurgical Society, May, 1902.

totally impracticable—especially with country physicians—and at least a small part of it entirely useless, if not harmful.

In the first place, I believe no man or woman should be permitted to handle a labor case who is not thoroughly acquainted with the anatomy of the female pelvis and its contents, and their relation to one another. He should also understand the processes of gestation—the relation of the mother and her child in utero—a high moral conception of his obligation to preserve the life of both, regardless of the wishes of any concerned or the sentiment of the public.

"Standing by a purpose true," he should be willing to dare and die, if need be, rather than swerve from the path of right and of honor.

If a man is thus qualified, then he may fit himself up for obstetric work. A good substantial leather bag of ample capacity; and in this he should keep a pair of forceps of some good make, strong curved needles, needle forceps, scissors, a card or two of strong silk, one or two soft rubber catheters, and a good syringe, antiseptic gauze, absorbent cotton, box of borated talcum, Kelly's cushion, at least one clean, freshly-laundered apron, 4-oz. bottle of chloroform, an ounce Squibb's ergot, one-half ounce Norwood's tinct. verat., a dozen or so capsules each containing five grains quinine, an ounce of solution chloral—two drachms chloral to ounce of water—an ounce carbolic acid, and a box of bichloride tablets (antiseptic). This pouch he should carry with him to every case of labor.

In addition to this, a second pouch or roll, containing a full set of obstetric instruments—long forceps, craniotomy forceps, placental forceps, perforator, blunt hook and crotchet and vectis—should be kept at the office, readily accessible to any one who may be sent for it.

Thus equipped, when called to a case of labor, the doctor should be cool and possessed, cautiously avoid everything in the way of speech or manner that would cause the patient or her friends to be excited. If a stranger to you, very quietly introduce yourself in as pleasant a manner as possible, and, if the suffering of the patient will admit of it, a good plan is to spend just a few minutes in pleasant conversation, not referring to your patient's condition until you have succeeded in convincing her that you are a gentleman; at the same time you may be observing the frequency of the pains, and by her facial expression be able to judge of their force. When ready to begin your examinations, first see that the bed upon which your patient is lying is properly prepared—a good mattress on good springs is the ideal bed for a lying-in woman. This should have two sheets, with a large oil cloth next the mattress, and some protection on top of the bottom sheet. I prefer a large Kelly cushion with sleeve apron. A good device I have seen used is a temporary wheat-bran mattress, which absorbs all the water and blood, and can be taken out after labor is over and disposed of as the family sees fit. Having the patient comfortably in bed, be sure to have plenty of hot water which has been boiled, and with hot water, soap and brush spend several minutes in scrubbing the hands, and scraping the finger nails, until you are morally sure they are surgically clean. At this point I believe it is good practice to make some examination of the external genitals—you can do this while you are examining the abdomen—to determine, if possible, the position and presentation of the fetus, and if any doubt as to their cleanliness, better give them a pretty thorough washing with hot water and soap, at same time frame some kind of reason for doing so that will not offend your patient or reflect upon her tidiness.

After satisfying yourself, if you can, by external manipulation, palpation, etc., as to the position and presentation of the fetus, again wash hands in soap and water, then bathe them in a solution of bichloride—one tablet to about a pint of water. I should have advised two basins, one for soap and water and one for bichloride solution. With hands thus prepared, and seated by the bed, introduce the index finger gently through the vulva, and on up to the mouth of the womb, observing, as you pass the finger up, the condition of all the parts—the hardness or laxness of the perineal muscles, and the vaginal wall, the sensitiveness of the parts, the condition of the mouth of the womb, whether dilated or to what degree, whether dilatable or hard and dense or thinned out to a feather-edge at opening and tense. At this examination—and I will say that very few examination of this kind should be made in any case of labor—be sure to verify or correct your diagnosis of position and presentation—that is, if the mouth of the womb is sufficiently dilated to do so, and at same time do not fail to observe the effect produced by each succeeding pain. When you have done all this, you may be justified in giving your patient such assurances as your findings will justify. In this stage of labor, if the position of the child and the presentation is favorable, and the pains are regular and effective, I believe it is well that the physician remain much of the time away from his patient, and out of the room, at same time be subject to her call. If the os is rigid and pains hard and ineffective, give her dose of chloral (fifteen grains) or one-fourth grain morphine, hypodermically, which will likely relax the system, and thereby hasten dilatation.

Indeed, during the first stage of labor, usually, there is little for the physician to do. It is better, usually, to let the sac rupture spontaneously, but I have found an occasional case in which the pains become ineffective, and would lose force just about the time when the second stage should set in, when by rupturing the thick dense sac the expulsive pains would begin and labor would advance to a finish.

At the beginning of the second stage it is my practice to make thorough digital examination to assure myself of the probable size of the child's head, and after observing a few pains to determine as to the probable necessity of using the forceps. As the head of the child descends and encroaches decidedly upon the soft parts and the perineal tissues made tense by its pressure, a good plan is to request the patient to refrain from bearing down, and with each pain, if the tension is very great, to make gentle upward pressure with the hand, and between the pains to endeavor to deliver the head. This can often be done by making downward pressure with left hand on abdomen above the womb, while with the right hand the soft parts are lubricated and worked backward over the prominence of the forehead of the child, when, of course, the tension is taken off and the head will be delivered without further trouble or danger. The head being delivered, attention must be given to the delivery of the shoulders. Holding the head in one hand, and at same time raising it to as high a point as is safe, the fingers of the other hand should be introduced in search for the arm lying posteriorily, and with finger in axila, by gentle traction, bring this arm down and out, and the work of the second stage is practically over.

In this stage of labor, particularly if the woman is nervous and the pains unsatisfactory, the inhalation of chloroform during the pains will often aid in quieting the nervousness, relaxing the parts, and promoting the expulsion of the head.

After the second stage has been completed ,notwithstanding the laity, and some doctors, look upon the remaining process as a very trivial matter, "only the taking of the after-birth," yet the longer I live the more my experience teaches me that this, the third stage of labor, is the most critical, frought with more danger to the woman than any other, or both the first and second stages combined. Indeed, I feel that this is so frequently true, that I would be willing to at least divide the fees with him who would relieve me of the responsibility of this stage of labor. However, after the child has been expelled and separated from the mother, the operator's hand should be placed upon the mother's abdomen, and if the uterus can be felt to contract firmly under the hand, all well; but, if not, a gentle kneading and grasping the womb with the hand will likely bring about contraction. If there is good contraction, and no immediate danger of hemorrhage, it is a good plan to wait ten or fifteen minutes, in order, first, that the womb may have time to regain its tone and energy, and, then, that the mouths of the intra-uterine vessels may be filled with coagula. After this few minutes rest, and being satisfied that the uterus is contracting properly, the placenta may be expelled by the expression method. My plan is to stand with my right side next the bed, and with the fingers of both hands dipped down well above and around the womb to make firm compression with each recurring contraction, and by this means express the after-birth out of the womb and out of the vagina. A little tact in doing this will often enable the operator to "clear" the womb perfectly with one or two efforts, and my practice is to continue squeezing the womb until I have gotten away every vestige of membrane that may be inclined to hang after the placenta has been expelled.

After clearing the womb, it is well to watch it closely for thirty minutes, and if any doubt about it contracting, the operator should keep it in the grasp of his hand, through the abdominal wall, until he is satisfied with its energy. When the mother is safe, attention should be given to the child. See if it is a perfect child, and finding it so, be sure to gladden the mother's heart by telling her it is so.

See that the bathing is properly done with warm water in a warm room, that the eyes are clean and in a healthy condition. When the bathing is done, be sure to dress the cord aseptically, and over the dressing place a liberal piece of absorbent cotton, and over this and around the body of the child apply a simple bandage about four inches wide, snugly but not tight. I have no patience with the practice of leaving the baby's cord undressed like a pig. After the baby is cared for, then attention should again be given to the mother. See that she is made comfortable. Have every vestige of soiled clothing and bed clothing exchanged for clean, and have all blood stains or other stains carefully wiped off her person, and if very nervous or suffering much pain give her one-fourth grain morphia, hypodermically.

Assuring himself that the womb is contracting properly, and mother and child doing well, the physician may take his departure, but with engagement to return within the next eight or ten hours—

this visit is simply to assure himself of the safety of his double charge, and at the same time should give directions as to nursing the child and feeding the mother, etc.

The next day after labor the vagina of the mother should be douched out with hot sterilized water, to which may be added a few drops of carbolic acid or creolin. This douching should be done by the physician himself, and will add to the comfort of the patient if done every day for three or four days succeeding labor.

DON'TS.

A doctor should never go to a case of labor without his pouch containing everything which he may need in emergency.

If possible to avoid it, never allow a woman to go into labor on a feather bed. Never attend a woman in labor with even a possible danger of infecting her; therefore, while treating erysipelas or any infectious disease, or after handling septic cases, a physician should avoid, if possible, attending labor cases. The tracks of a careless doctor may sometimes be traced by his frequently having cases of puerperal sepsis, and, what to the patient and her friends, perhaps, may appear accidental, is, in truth, the fault of the careless doctor. For the same reason a doctor should never touch the vulva of a laboring woman until he has thoroughly scrubbed his hands and arms to his elbows, scraped his finger nails and then bathed them in a solution of bichloride; and don't use a towel or anything to dry the hands until this examination is done, then wash the hands with soap and water, and dry with clean towel, and don't fail to wash the hands and bathe in bichloride before each vaginal examination.

Don't become impatient in first stage of labor because the advancement is not rapid—nature's methods are usually the best, but if the recurring pains are ineffective, not causing the mouth of the womb to dilate easily, and are telling on the patient's strength, better give her a pretty full dose of chloral or morphine and rest the tired creature for a while; but don't endeavor to open the canal by force, unless an emergency, such as flooding or eclamptic convulsion, demands it. A slow process in first stage is better than a torn cervix. Don't repeat vaginal examination frequently, nor keep the hand or finger within the vagina but a few miutes at a time, except for reasons which may make it necessary. These examinations tend to dry the secretions which nature provides for lubricating the parts, and are calculated to irriate the parts and actually, in many instances, to retard labor.

Of course, always be courteous to your assistants, but endeavor to impress upon them the fact that you are the doctor, and never allow them to dictate to the patient how she should lie upon the bed, or how she should hold her head or her hands, or how she must "bear down and help the pains along." Their suggestions are often worse than nonsense, and you are there to know what is best for the patient. Never treat a complaint by the patient in labor, that she has a bad headache, lightly—it may mean something grave later on. Never leave the patient's house, or get out of hearing of her call after expulsive pains are established, or the sac has been ruptured.

Don't be hasty to apply forceps, but don't be afraid to do so when necessity requires—that is, don't allow the woman to go on through hours of agonizing, ineffective pain when she may be harmlessly relieved in a few minutes by using the forceps.

Don't encourage the woman to "bear down" if the expulsive pains are vigorous, when the soft parts at the outlet are made very tense by the child's head passing through them—better insist on her to refrain from bearing down with the pain, and in the interval between the pains to introduce two or three fingers into the vagina at its posterior angle and press back firmly the posterior commissure, and at same time grasp the head with the fingers of right hand behind and the fingers of the left hand in front and make traction downward and forward; or else slip the blades of a pair of thin, delicate, short, forceps around the head and make gentle traction, lifting the head as much as possible from the posterior aspect—all this to be done between the pains. By thus taking care, you may save may a tear.

After the child is born, don't neglect to make it cry.

Don't attempt to deliver the after-birth until fifteen to thirty minutes after child is born, except for urgent reasons; at same time don' neglect, during the interval, to keep close watch over the womb to know that it is regularly contracting. Don't make traction by the cord to deliver placenta—you can do it much easier and quicker and more perfectly by the expression method.

While waiting for the fifteen or thirty minutes to deliver the after-birth, don't forget or neglect to dress the baby's cord antiseptically, and as dry as possible, and don't forget or neglect to have binder put on babe.

When the after-birth has been delivered, don't neglect to keep the womb in the grasp of the hand, through the abdominal wall, for several minutes to insure proper contraction.

Don't allow the woman's clothing changed immediately, especially if she is much exhausted; but never allow her to remain more than a few hours without changing and thorough cleansing.

Don't put the babe to the breast for an hour or so—until the mother has rested a while.

Don't neglect to see that the babe's eyes are properly cleansed—ordinarily a solution of boracic acid is sufficient for this purpose.

Don't fail to douche the vagina in about twenty-four hours after labor, and do it yourself with proper aseptic precautions. I believe it is, as a general thing, hazardous to delegate this duty to the nurse.

Don't fail to do your whole duty, from start to finish, and then don't fail to charge for your services a liberal fee, and collect your bill, and you are then assured of a life-long friend in the person of your patient.

